# Quest for Orthologs in the Era of Biodiversity Genomics

**DOI:** 10.1093/gbe/evae224

**Published:** 2024-10-15

**Authors:** Felix Langschied, Nicola Bordin, Salvatore Cosentino, Diego Fuentes-Palacios, Natasha Glover, Michael Hiller, Yanhui Hu, Jaime Huerta-Cepas, Luis Pedro Coelho, Wataru Iwasaki, Sina Majidian, Saioa Manzano-Morales, Emma Persson, Thomas A Richards, Toni Gabaldón, Erik Sonnhammer, Paul D Thomas, Christophe Dessimoz, Ingo Ebersberger

**Affiliations:** Department for Applied Bioinformatics, Institute of Cell Biology and Neuroscience, Goethe University, Frankfurt, Germany; Institute of Structural and Molecular Biology, University College London, WC1E 6BT, London, UK; Department of Integrated Biosciences, The University of Tokyo, 277-0882 Tokyo, Japan; Barcelona Supercomputing Center (BSC-CNS), 08034 Barcelona, Spain; Institute for Research in Biomedicine (IRB Barcelona), The Barcelona Institute of Science and Technology, 08028 Barcelona, Spain; SIB Swiss Institute of Bioinformatics, 1015 Lausanne, Switzerland; Department of Computational Biology, University of Lausanne, 1015 Lausanne, Switzerland; Department of Comparative Genomics, Institute of Cell Biology and Neuroscience, Goethe University, Frankfurt, Germany; Department of Genetics, Harvard Medical School, Boston, MA 02115, USA; Drosophila RNAi Screening Center, Harvard Medical School, Boston, MA 02115, USA; Centro de Biotecnología y Genómica de Plantas, Universidad Politécnica de Madrid (UPM) - Instituto Nacional de Investigación y Tecnología Agraria y Alimentaria (INIA-CSIC), Campus de Montegancedo-UPM, Madrid, Spain; Centre for Microbiome Research, School of Biomedical Sciences, Queensland University of Technology, Translational Research Institute, Woolloongabba, Queensland, Australia; Department of Integrated Biosciences, University of Tokyo, 277-0882 Tokyo, Japan; SIB Swiss Institute of Bioinformatics, 1015 Lausanne, Switzerland; Department of Computational Biology, University of Lausanne, 1015 Lausanne, Switzerland; Barcelona Supercomputing Center (BSC-CNS), 08034 Barcelona, Spain; Institute for Research in Biomedicine (IRB Barcelona), The Barcelona Institute of Science and Technology, 08028 Barcelona, Spain; Department of Biochemistry and Biophysics, Stockholm University, Science for Life Laboratory, Solna, Sweden; Department of Biology, University of Oxford, Oxford, OX1 3SZUK; Barcelona Supercomputing Center (BSC-CNS), 08034 Barcelona, Spain; Institute for Research in Biomedicine (IRB Barcelona), The Barcelona Institute of Science and Technology, 08028 Barcelona, Spain; Catalan Institution for Research and Advanced Studies (ICREA), Barcelona, Spain; CIBER de Enfermedades Infecciosas, Instituto de Salud Carlos III, Madrid, Spain; Department of Biochemistry and Biophysics, Stockholm University, Science for Life Laboratory, Solna, Sweden; Department of Population and Public Health Sciences, University of Southern California, Los Angeles, CA, USA; SIB Swiss Institute of Bioinformatics, 1015 Lausanne, Switzerland; Department of Computational Biology, University of Lausanne, 1015 Lausanne, Switzerland; Department for Applied Bioinformatics, Institute of Cell Biology and Neuroscience, Goethe University, Frankfurt, Germany; LOEWE Centre for Translational Biodiversity Genomics, 60325 Frankfurt, Germany; Senckenberg Biodiversity and Climate Research Centre (S-BIK-F), Frankfurt am Main, Germany

**Keywords:** ortholog search, annotation transfer, domain architecture, protein structure, FAIR, noncoding RNA

## Abstract

The era of biodiversity genomics is characterized by large-scale genome sequencing efforts that aim to represent each living taxon with an assembled genome. Generating knowledge from this wealth of data has not kept up with this pace. We here discuss major challenges to integrating these novel genomes into a comprehensive functional and evolutionary network spanning the tree of life. In summary, the expanding datasets create a need for scalable gene annotation methods. To trace gene function across species, new methods must seek to increase the resolution of ortholog analyses, e.g. by extending analyses to the protein domain level and by accounting for alternative splicing. Additionally, the scope of orthology prediction should be pushed beyond well-investigated proteomes. This demands the development of specialized methods for the identification of orthologs to short proteins and noncoding RNAs and for the functional characterization of novel gene families. Furthermore, protein structures predicted by machine learning are now readily available, but this new information is yet to be integrated with orthology-based analyses. Finally, an increasing focus should be placed on making orthology assignments adhere to the findable, accessible, interoperable, and reusable (FAIR) principles. This fosters green bioinformatics by avoiding redundant computations and helps integrating diverse scientific communities sharing the need for comparative genetics and genomics information. It should also help with communicating orthology-related concepts in a format that is accessible to the public, to counteract existing misinformation about evolution.

**Significance**

The identification and analysis of orthologs play a crucial role in evolutionary research, especially in the rapidly advancing field of biodiversity genomics. In this review, we highlight recent advancements in orthology-based research and propose strategies for addressing current limitations. A comprehensive understanding of existing methods and open challenges is essential for utilizing orthology assignments effectively in state-of-the-art biodiversity genomics research.

## Introduction

Biodiversity loss is listed as one of the five most pressing global risks by the World Economic Forum (https://www.weforum.org/publications/global-risks-report-2023/). Estimated consequences include the alteration of food webs (Pilling et al. 2020), changes in the metabolic pan-network of an ecosystem through the extinction of species (Cannell et al. 2020), and, ultimately, the potential collapse of entire ecosystems. Investigating the causes and consequences of biodiversity loss has substantially intensified over the past years. Since the study of biodiversity loss increasingly involves the comparative analysis of biological sequences at the genome scale, it has become a focal area of “Biodiversity Genomics” (Supple and Shapiro 2018). As a common principle, Biodiversity Genomics investigates how sequences and the functions they convey have changed over time, which sequence variants are present in what taxa, and which evolutionary regimes have shaped this process. On this basis, the presence, the abundance, and the genetic diversity of species in an ecosystem can be monitored over evolutionary time (Leigh et al. 2019). Additionally, biological sequences help to mine the hitherto only marginally tapped wealth of natural products via, e.g. the identification of novel biosynthetic gene clusters (Marcet-Houben et al. 2023).

**Fig. 1.**
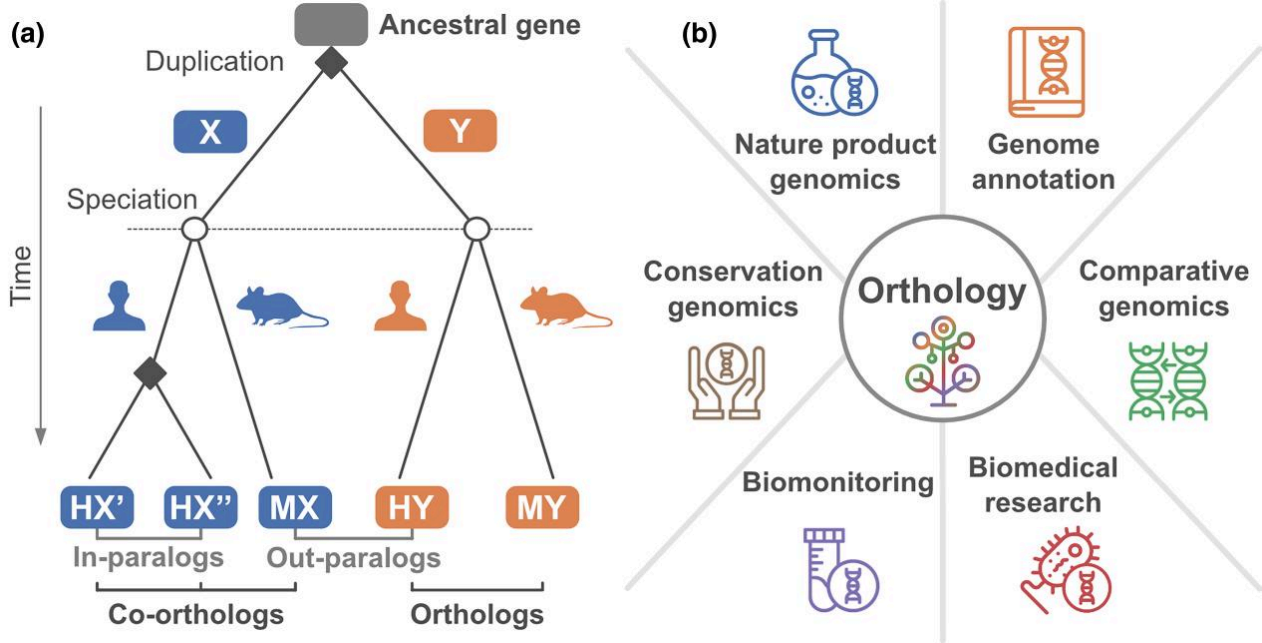
Biodiversity genomics are rooted in ortholog identification. a) The evolutionary concepts of orthology and paralogy. The evolutionary lineages of genes sharing the same ancestry (homologs) are split either by gene duplication events (square nodes), giving rise to paralogs, or by speciation events (circle nodes), resulting in orthologs. The depicted tree represents a scenario where a gene duplication prior to the speciation event gives rise to the out-paralogous genes X and Y that reside in the human and mouse lineages, respectively. The split of the human (H) and mouse (M) lineages resulted in the orthologous groups HY and MY. In the case of gene X, a subsequent gene duplication in human formed the in-paralogous gene pair HX′ and HX″ where both human genes are co-orthologous to MX. Pictograms provided by PhyloPic. b) The identification of orthologs forms the foundation for gene annotation transfer between species, because they represent our best inferences of genes with corresponding functions. Together with de novo annotation methods, orthologs are needed to annotate comprehensive catalogues of genes present in an organism (Genome annotation). Consequently, any analysis that compares which functions (i.e. genes) are available in different genomes is also rooted in orthology (Comparative genomics). Identifying orthologs that are specific to a taxonomic group can help to identify pathogenicity-related factors (Biomedical research) or to define genetic markers hat help in sequence-based species identification (Biomonitoring). They inform how robustly a molecular function is represented in an ecosystem (Conservation genomics), and they can identify novel gene clusters that produce secondary metabolites (Natural product genomics). All symbols provided by Icon Market from Noun Project.

“Biodiversity Genomics” has significantly benefited from the ease with which sequences of even very large genomes can be assembled. This has resulted in the rapid accumulation of publicly available genome sequences that represent the tree of life with increasing coverage. The integration of these novel genomes into Biodiversity Genomics studies essentially depends on the accuracy with which the evolutionary relationships of the compared sequences can be determined. Biological sequences that share a common ancestry (homologs) can be categorized based on the evolutionary event they originated from (Fig. 1a). Orthologs emerge as a consequence of a speciation event, whereas paralogs emerge via gene duplications (Fitch 1970). Consequently, paralogous sequences from two species are evolutionarily more distantly related than the corresponding ortholog pairs. It is for this reason that orthologs are often loosely referred to as the “corresponding” genes in two species and often share the same or at least similar function (Gabaldón and Koonin 2013). This makes the identification of orthologs the basis of many Biodiversity Genomics analyses (Fig. 1b).

**Fig. 2.**
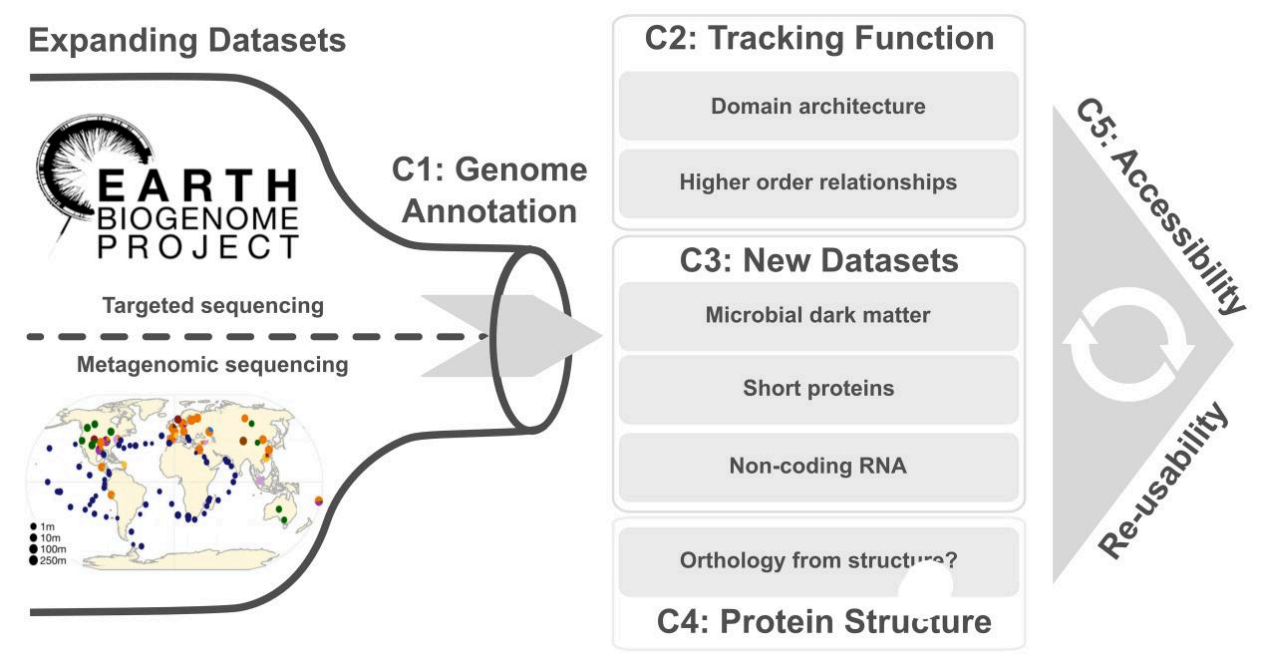
Current challenges of ortholog-based research. The logo of the EBP is used to symbolize large, targeted sequencing projects. The world map in the bottom half shows the sampling distribution of 13,174 metagenomes from 14 habitats as an example of a large-scale metagenome project [Figure adapted from Coelho et al. (2022)]. The bottle shape represents the large number of available datasets which present several new challenges. As the first challenge, all new datasets must pass through the genome annotation bottleneck (C1). Challenges regarding technical aspects of orthology prediction are presented in boxes C2-4. Finally, orthology assignments should be FAIR (C5) (Wilkinson et al. 2016).

Since 2009, current challenges in the field of orthology inference have been discussed and addressed by the Quest for Orthologs (QfO) Consortium. Since its inception, the QfO Consortium successfully established a regularly updated benchmark service for orthology predictions (Nevers et al. 2022), curating and maintaining a set of reference proteomes (https://www.ebi.ac.uk/reference_proteomes). It has been also advancing the state of the art of orthology-related applications for more than 10 years (Linard et al. 2021). The QfO Consortium gathered for its seventh on-site meeting on 2022 September 17 to 18 in Sitges, Spain, in conjunction with the 21st European Conference on Computational Biology (ECCB 2022). Using key points from this meeting as anchor points, we will here review challenges and future directions for orthology inference. We will use the expanding datasets in the biodiversity genomics era to motivate the relevance of these issues (Fig. 2). The almost exponentially growing number of newly sequenced genomes results in an annotation bottleneck that needs to be addressed at an appropriate scale (Challenge 1). It is further essential to bridge the gap between potential and actual knowledge that can be derived from this flood of data. To do this, the resolution of orthology prediction must be increased to the protein domain level to better track the change of gene function through time (Challenge 2). Additionally, the analysis of large genome collections should extend beyond standard protein-coding genes (Challenge 3). With machine learning-driven predictions of protein structures being readily available, it will be necessary to explore how the comparison of protein structures can help to identify distantly related orthologs whose sequences are no longer more similar than expected by chance (Challenge 4). Finally, efforts of the ortholog community should be made more accessible to scientists of all communities (Challenge 5) and redundant computations should be minimized to reduce the collective computational carbon footprint (Grealey et al. 2022). Each of these challenges will be expanded upon in separate sections below.

## Expanding Datasets Create Opportunities and Challenges

### Cataloguing Biodiversity With Targeted Sequencing

The scope of large-scale sequencing projects has expanded rapidly in recent years. One manifestation of this is the Earth Biogenome Project (EBP)—a “moonshot” project that aims to sequence all 1.6 million taxonomically classified eukaryotic species (Lewin et al. 2022). As of January 2024, 2,313 species from 24 phyla have been sequenced, assembled, and made publicly available under the umbrella of the EBP (https://goat.genomehubs.org/projects/EBP), and this number is growing rapidly. Many of these genome sequences have been contributed by associated projects, each of which targets a specific group of species. This includes the vertebrate genome project (https://vertebrategenomesproject.org/) which aims to sequence over 260 different vertebrate genomes in its first phase and the Moore Foundation Aquatic Symbiosis project (https://www.aquaticsymbiosisgenomics.org/) which targets hundreds of symbiotic organisms (e.g. corals, sponges, lichens, algae, and protists). The Darwin Tree of Life (DToL) project, as another example, aims to sequence all eukaryotic species in Britain and Ireland and increasingly tackles more challenging genome assembly projects. Recently, DToL celebrated its 1,000th genome sequence assembly, the European mistletoe (*Viscum album*) whose genome is 30 times larger than that of humans. Current large-scale sequencing efforts are committed to the production of chromosome-level, reference-grade assemblies—an important foundation for obtaining comprehensive gene catalogues with little missing data due to assembly gaps. This helps to minimize artifacts in downstream orthology prediction steps (see Challenge 1). With corresponding genome announcements written in a semiautomatic manner, the data are made publicly available and monitored in real time via the Genome on a Tree (GoaT) project (Challis et al. 2023). Thus, the status of the EBP provides a glimpse of the data scale and assembly quality that will become available in the upcoming years. In the long run, these projects will allow us to chart a large part of the genomic diversity on Earth.

### Metagenomic Sequencing Unearths the Diversity of Prokaryotic Communities

Catalogues of prokaryotic diversity are expanding quickly, with huge datasets emerging from metagenomic sequencing of diverse environments (Laiolo et al. 2024). Metagenomic analyses are no longer used only for diversity tag or barcode sequencing, but to assemble genomes directly from metagenomic reads (Pasolli et al. 2019). Metagenome-assembled genomes (MAGs) are key for evaluating the ecosystem service that is provided by different microbial communities (Grossart et al. 2020). MAGs can also help to compare taxonomic and functional profiles in an ecosystem, for example, by identifying redundant metabolic functions present in multiple taxonomically distinct organisms (Louca et al. 2018).

One example for large-scale metagenomic sequencing efforts is the Global Microbial Gene Catalogue across 13,174 metagenomes from 14 habitats. To date, it includes 303 million nonredundant genes (Coelho et al. 2022). Functionally annotating such large-scale datasets requires specialized algorithms like the eggNOG-mapper v2 that is optimized for fast database queries and I/O operations (Cantalapiedra et al. 2021) or the MMseqs2 suite. MMseqs2 quickly assigns contigs with taxonomic labels by concentrating on sequence fragments that share a minimum level of similarity to sequences in the reference database and removes redundant protein sequences by linear scaling clustering (Mirdita et al. 2021). However, removing redundancy also means potentially losing information. This can become an issue for downstream analyses, especially since bacterial research communities increasingly focus their investigations on intraspecies variations (Iruegas et al. 2023).

The rapid accumulation of genomic data from both targeted and metagenomic sequencing poses a challenge that outpace strategies devised at a time when sequencing a single genome was a major feat. Consequently, databases, publications, and the way we investigate and report gene family evolution must be adjusted to cope with this flood of data.

## Challenge 1: Ortholog Search Across Tens of Thousands of Genomes

Less than 20 yr ago, genome-based research was possible for only a few model organisms (e.g. *Homo sapiens*, *Drosophila melanogaster*, *Caenorhabditis elegans*, and *Saccharomyces cerevisiae*), but it became quickly obvious that both accuracy and resolution of comparative genomics analyses benefit substantially from a more comprehensive taxon sampling (Putnam et al. 2007). As the bottleneck imposed by lack of genome assemblies ceases to exist, comparing genomic sequences themselves is now often only of moderate interest. Instead, most analyses are concerned with lineage-specific changes in gene content (Ocaña-Pallarès et al. 2022), transposon activity (Martelossi et al. 2023), or horizontal gene transfer (Irwin et al. 2022), to name a few. All these analyses require accurately inferred protein-coding genes for the respective genome assemblies, and artifacts introduced during this process can generate spurious signals that confound the downstream conclusions (Bálint et al. 2024). The scarcity of genome availability, once a major bottleneck, has now been superseded by the challenge of functionally annotating tens of thousands of genome assemblies and inferring patterns of gene evolution—an endeavor for which understanding orthology relationships is essential.

State-of-the-art methods of gene prediction integrate intrinsic sequence information like coding-potential and splice site motifs with extrinsic data (Gabriel et al. 2023). Genes with homologs in other species can be inferred with information from related organisms (Bruna et al. 2024). Complementary to the evolutionary approach, genes can be predicted with the help of transcriptomic data (e.g. Hoff et al. 2016). This allows for the detection of evolutionarily young genes that lack homologs in public databases, and of genes that evolve so quickly that the homology inference fails (Jain et al. 2019). However, genes that are lowly expressed or have a particular spatial or temporal expression pattern are likely to be missed. Mitigating these limitations is necessary for cataloguing the full breadth of sequences that make up life on Earth, but generating these data for all assembled genomes is not feasible. Therefore, Guigó (2023) has recently proposed a hierarchical sequencing scheme. In brief, this scheme suggests assembling the genome of one organism per taxonomic class to high contiguity and additionally constructing a comprehensive, whole-body, long-read RNA-seq cell atlas. Further taxa nested in this class can then be sampled with decreasingly elaborate sequencing efforts. However, even if transcriptomic data become available for one representative species per genus, it will still be necessary to project gene predictions and their functional annotation to ∼10 times more species.

### Quality Control of Genome Annotation

In ortholog-based research, common sources of artifacts include genes situated in assembly gaps that can be mistaken for gene loss. Genes identified only over part of their length, or the failure to differentiate between intergenic regions and introns can falsely indicate changes in gene structure. Additionally, an incomplete masking of e.g. transposons can lead to artificially inflated sets of protein-coding genes. Future studies should therefore determine whether the choice of annotation methods has a significant impact on downstream orthology inference. To what extent a gene set can be considered complete has been most addressed with the BUSCO completeness score (Manni et al. 2021). In brief, BUSCO relies on curated sets of single-copy orthologs that are conserved in predefined taxonomic groups. Orthologs for each member of these sets are identified and are labeled as “complete,” “fragmented,” or “missing” according to a length criterion. This general idea was recently extended with two new approaches that are not restricted to single-copy orthologs and hence query more marker genes than BUSCO. fCAT (https://github.com/BIONF/fCAT) assigns completeness labels not only based on gene length but also takes the expected feature protein architecture similarity score into account (Dosch et al. 2023). In contrast to BUSCO, fCAT can utilize custom sets of core genes that can be curated from any source of orthology assignments and can cover any taxonomic range. OMArk evaluates the completeness of proteomes by rapidly assigning query protein sequences to a set of evolutionarily conserved hierarchical orthologous groups (HOGs) for a taxonomic level (Nevers et al. 2024). Additionally, OMArk identifies proteins with no known homologs or with taxonomically unexpected orthologs in the target proteomes. Those may derive from fast-evolving or hitherto undiscovered gene families but can also indicate spurious gene models—an aspect typically ignored by other tools. Proteins with an unexpected taxonomy distribution may also be caused by contamination and are flagged as a contaminant by OMArk if more orthologs to it are found outside of the target assembly's lineage than expected by chance. Nevers et al. (2024) identified 72 proteomes from UniProt that are contaminated with sequences mostly of bacterial or fungal origin. This finding highlights the need for more stringent quality control during the curation of reference proteomes, and the need for reference-grade assemblies, a stated ambition of many current large-scale biodiversity genome diversity projects.

While contaminations from nontarget organisms do increase spurious orthology assignments in comparative analyses (Bálint et al. 2024), these contaminants may not be devoid of useful information. Indeed, recent studies do not only explore contaminations in genome assemblies to find residues of sample preparation (Chrisman et al. 2022), but have used them, e.g. for reconstructing the microbiome of a target species (Foo et al. 2023). To harness this unused potential, scalable approaches for the identification and analysis of nontarget sequences in newly sequenced assemblies are needed.

### Computational Limitations of the Ortholog Search

Graph-based orthology inference methods scale quadratically with the number of sequences investigated (Altenhoff et al. 2019), making computational time a bottleneck for analyzing large datasets. It is therefore not surprising that even the latest releases of ortholog databases cover only up to 2,000 out of over 15,000 eukaryotic assemblies in the International Nucleotide Sequence Database Collaboration (INSDC) (Arita et al. 2021; Kuznetsov et al. 2023). To close this gap, multiple approaches were recently developed. For example, SonicParanoid2 reduces the runtime of orthology inference using machine learning (Cosentino et al. 2024). In brief, given a proteome pair *A* and *B* in which orthologs can be identified using a reciprocal similarity-based search, the search direction (A vs. B or B vs. A) with a shorter execution time is predicted using Adaptive Boosting. The “faster” search direction is then performed first, and based on its results, only those proteins that are likely to have an ortholog partner are considered in the second search. A benchmark revealed a speedup of the ortholog search by up to 42% while maintaining high precision and recall. Another method involves targeted profile-based searches that reduce the runtime to linear complexity, facilitating the identification of orthologs across thousands of taxa (Birikmen et al. 2021). Additionally, k-mer distance-based preclustering can be used to remove allelic variants and duplicates (Derelle et al. 2020). Dealing with the growing amount of incoming data will require even more efforts to reduce the computational burden. In this context, the carbon footprint associated with computational analysis has become a serious concern (Grealey et al. 2022), and it is important not only to optimize algorithms but also to avoid redundant analyses (see Challenge 5).

### Ortholog Search in Unannotated Genome Assemblies

Thus far, all ortholog search algorithms depend on the availability of a comprehensively identified gene sets for a genome assembly. However, the gene prediction process itself benefits from the information about orthology relationships as well, indicating that both tasks can be integrated. Tool to infer Orthologs from Genome Alignments (TOGA) facilitates a computationally efficient and comprehensive projection of genome annotation across species using whole-genome alignments (Kirilenko et al. 2023). Graph-based ortholog detection exploits that a sequence in one organism tends to look more like its ortholog in a second species than like a paralog. TOGA extends this similarity principle to the genomic context of a gene. For each annotated gene in a reference species, TOGA first infers the orthologous locus (or loci in case of co-orthologs). It then determines the positions of coding exons in each orthologous locus, which provides a comprehensive, high-quality annotation of genes conserved between reference and target species. TOGA scales linearly with the number of genomes and can therefore be used for hundreds of species. For example, TOGA was applied with human and mouse as references to 488 placental mammals and with chicken as a reference to 500 birds. However, the use of TOGA is limited to closely related species for which even intronic and intergenic parts of the genomes can still be aligned in a meaningful way.

Many research questions are centered around a specific set of genes and do not necessarily require an annotation of the complete genome. In such use cases, the novel tool fDOG assembly can perform orthology inference for genes of interest in unannotated genome assemblies (Collins et al. 2023). fDOG assembly extends existing orthologous groups by first identifying the region in the unannotated assembly that is most likely to harbor an ortholog. It then annotates genes in these regions using either AUGUSTUS which is guided by block profiles generated from existing orthologous groups, or MetaEUK. Any identified genes are then tested if they can be added to the orthologous group with a reciprocal best similarity search hit criterion.

In a time where sequencing of new genomes rapidly progresses, these methods mentioned above provide a strategy to cope with the gene prediction and orthology inference bottleneck. The decision of whether a full reference-based inference of all genes or a targeted ortholog search for a specific gene set is more fitting will then ultimately depend on the research question and the annotation status of a given dataset. Nevertheless, one could legitimately ask whether including all available genomic data is necessary to infer orthologous relationships. Instead, it might be more practical to down-sample available assemblies, trying to maximize the taxonomic diversity in the dataset (Bonnie et al. 2024). Future studies should therefore also aim at investigating more closely how much information is gained by the addition of data to different phylogenomic applications.

## Challenge 2: Tracking Functional Changes of Orthologs

Orthologs are typically considered the best guess for identifying functionally equivalent genes in two species. However, the probability of functional diversification increases over evolutionary time. While it is hard to trace the conservation of function in silico, information can be gathered that indicates a change of function.

### Increasing the Resolution to Feature Architecture Level

Amino acid sequence divergence alone is a poor proxy for the functional divergence of orthologs (Laurent et al. 2020). This opens the case for adding additional layers of information that aid to infer functional divergence of orthologs. One option is to annotate protein sequences with features that have more direct links to molecular function such as Pfam and SMART domains, signal peptides, transmembrane domains, or even low complexity regions. By now, many orthology databases provide the resulting feature architectures of the identified orthologs as accessory information (Altenhoff et al. 2021; Kuznetsov et al. 2023; Persson and Sonnhammer 2023). However, their comparison is left to the individual user, and the tracing of feature architecture changes across orthologs as an indicator of functional change is tedious. Dosch et al. (2023) simplified this task with FAS, a software that captures the pairwise feature architecture similarity between proteins as a score between 0 (no similarity) and 1 (identical). As a key innovation, a score maximization algorithm identifies the highest scoring linear path through redundant parts in a protein's feature architecture, e.g. resulting from overlapping annotations of Pfam and SMART domains. The method was applied to identify different variants of a bacterial pilus tip, indicating that different strains within the same species vary in the way they interact with the environment and/or their human host (Iruegas et al. 2023).

While FAS is a significant step toward the automated comparison of protein feature architectures and inferring functional divergence of orthologs, the scoring scheme remains ad hoc. Changes in protein feature architecture over evolutionary time can now be modeled with the tool DomArchov that exploits nonrandom constraints of multi domain architecture evolution (Cui et al. 2022). Using this software, it is now possible to simulate a range of protein feature architectures, compare observed changes in architectures over time, and determine whether these changes are significantly larger than expected.

Both FAS and DomArchov indicate that the conventional view of multidomain proteins as a single evolutionary unit during the ortholog search is helpful but sometimes misleading. While it simplifies the data processing and analysis considerably, it will provide spurious results if not all domains of a protein have the same evolutionary history (Persson et al. 2019). A few domain-based approaches to orthology prediction have been developed over the years, for example, SonicParanoid2 (Cosentino et al. 2024) or the methods used during construction of the prokaryotic COGs (Galperin et al. 2021) and MBGD (Uchiyama et al. 2019) databases. Recently, the InParanoiDB database was redesigned to contain both full-length and domain orthologs (Persson and Sonnhammer 2023). For the latter, the Domainoid algorithm (Persson et al. 2019) was applied to 640 eukaryotic and prokaryotic proteomes. The orthologous domains can then be used, e.g. to detect orthologous relations that are not found at the full-length level, or to extract discordant domain orthologs, where different domains have different evolutionary histories. InParanoidDB also provides a graphical display of the domain architectures in an ortholog group that allows domain searching and switching between full-length and domain ortholog groups.

### Higher-Order Relationships of Gene Families

Functional annotation transfer is a prime application of orthology assignments. Many studies accomplish this by connecting their proteins of interest to manually curated groups of functionally annotated orthologs provided, e.g., by KEGG (Kanehisa et al. 2023), COG (Galperin et al. 2021), or Panther (Thomas et al. 2022). However, it has recently been shown that the complementary use of paralogs for transferring functional annotation can increase the amount of transferable information (Stamboulian et al. 2020). This is one objective of the Phylogenetic Annotation using Gene Ontology (PAN-GO) project (Gaudet et al. 2011). PAN-GO integrates experimental knowledge of gene functions across entire gene family trees, including both orthologs and paralogs, to create precise and comprehensive descriptions of gene functions. Using extensive automated functional annotation integrated with expert curation, the PAN-GO project has created models of function evolution across nearly 10,000 gene families, covering over 82% of human genes (Aleksander et al. 2023). PAN-GO annotations are available as part of the PANTHER database. They serve as a knowledge base of protein function that has been continually expanded since its initial release (Thomas et al. 2022). In addition to providing a comprehensive catalogue of gene functions, this work highlights again that a large fraction of our knowledge derives from experimental studies of homologs in model organisms like the mouse, fruit fly, *C. elegans*, and yeast.

Even with manual curation in place, the accuracy of the functional annotation transfer increases with decreasing phylogenetic distances between the compared species. This is because orthologs having less time to functionally diverge. This calls for a comprehensive set of functional studies from experimental assays in taxonomically diverse species. Paired with more sophisticated computational methods to detect functional diversification between orthologs (see Challenge 2), this can help to alleviate the burden of manual curation. Generating these datasets will also add to the pool of training data that is available to existing tools for the prediction of gene function directly from sequence data (Kulmanov et al. 2018).

### Coevolution as an Indication of Functional Interdependence

Phylogenetic profiling studies identify functionally interacting proteins by comparing presence–absence patterns across large taxon collections (Dembech et al. 2023). Traditional phylogenetic profiling methods have been limited by the assumption of uniform correlation in coevolving proteins across all species (Moi and Dessimoz 2023). This assumption, effective for interactions common to ancestral lineages, struggles to capture lineage-specific interactions. To mitigate this, a novel approach employs graph neural networks (GNNs) to incorporate the species tree's structure and provide insights into the temporal and taxonomic emergence of these interactions (Moi and Dessimoz 2022). Such an approach will be particularly relevant to mine the wealth of biodiversity genomes discussed above. The ability of GNNs to predict when and in which taxa certain interactions appeared complements the PAN-GO project's efforts in creating detailed interaction models of function evolution across gene families. Fusing evolutionary data with interaction and functional analysis promises to increase the accuracy and predictive power of functional annotations based on orthologs and paralogs.

## Challenge 3: Orthology Inference off the Beaten Track

In the past decade, the focus of comparative genomics was directed on protein-coding genes that can be annotated with sequence-based methods. These genes are consequently well covered in established orthology databases, but compiling a catalogue of all genes must also include those that are more difficult to annotate (Amaral et al. 2023). One must therefore not only process the amount of data that is becoming available, but also adapt existing methods to infer orthologs beyond standard proteomes.

### Orthology of Short Proteins

The length distribution of annotated proteins is surprisingly uniform across the tree of life, with a high proportion of sequences ranging between 50 and 500 AA (Nevers et al. 2023). There is no consistent terminology, but proteins shorter than 100 AA (and particularly those shorter than 50 AA) are referred to as small- or microproteins (Storz et al. 2014). Microproteins are abundant across all domains of life and account for 3% to 5% of a species’ proteome (Pueyo et al. 2016). However, they have so far flown under the radar of most comparative analyses for two main reasons. First, the corresponding genes are hard to annotate. This results in a high number of spurious gene predictions that prohibitively inflate the computational burden of orthology assignments (Storz et al. 2014). Experimental data like ribosomal profiling or mass spectrometry can provide more direct evidence for the annotation of microprotein-encoding genes (Kute et al. 2021). Unfortunately, the generation of such data does currently not scale with the growing number of available genome sequences. Second, it is challenging to identify orthologs of microproteins. Many of them may be evolutionarily very young and are thus only present in a narrow taxonomic range because the corresponding genes are more likely to be created de novo from random noncoding DNA than larger genes (Montañés et al. 2023). In addition, they evolve more rapidly than genes that encode longer proteins (Slavoff et al. 2013). These factors, together with their short length, which limits the amount of phylogenetic signal that is necessary to resolve their evolutionary relationships, make the detection of orthologs challenging (Jain et al. 2019). It is therefore particularly hard to distinguish between failing to sample (recover) an ortholog of a microprotein and its genuine absence. Tracking the full genetic biodiversity on Earth will consequently require the development of specialized applications for detecting orthologs to small proteins. This will not only help with the annotation transfer of short genes but also with shedding light on their evolution.

### Novel Bacterial Gene Families

Insights from a recent large-scale metagenomic effort on the global microbiome (Coelho et al. 2022) indicate that there are many novel gene family clusters whose biological relevance is still unknown. Recent estimates indicate that about 25% to 50% of all observed environmental genes have no significantly similar sequences in the current databases, leaving their function elusive (Coelho et al. 2022). A recent study discovered novel orthologous groups (OGs) of high functional evolutionary significance from 140,000 prokaryotic MAGs of uncultivated taxa (del Río et al. 2024). The authors identified more than 400,000 protein-coding gene families found exclusively in uncultivated taxa that are missing from current databases. Still, several criteria indicate their functional relevance: they all show evidence of strong purifying selection, span multiple species, and contain a conserved protein region of at least 20 amino acids. Many of these novel OGs were functionally annotated by using structural alignments and/or mapped to highly conserved genomic locations, and they could be linked to important biological processes such as central metabolism, defense systems, and cell motility (del Río et al. 2024). Furthermore, hundreds of these novel OGs were identified as synapomorphies for high taxonomic ranks, highlighting their potential as lineage-defining traits contributing to the evolution and diversification of these uncultivated lineages.

Notably, the same study argues that these novel OGs cover only ∼5% of all unknown sequences, the rest being discarded due to quality filters or lack of supporting data. Thus, this new set of OGs (https://novelfams.cgmlab.org) may only represent the tip of the iceberg and the comparative microbial genomics community will soon face the challenge of an exponential increase in new genes, families, and orthologs. One approach to tackle the challenge of assigning tentative functions exploits the embedding of genes into their genomic context composed by neighboring genes. For example, genes that tend to conserve their gene order more than expected over evolutionary timescales, for example, through consistent co-association within operons (Rocha 2006), have been used to extend functional assignments to previously undescribed genes (Djahanschiri et al. 2022). Moreover, deep learning approaches have successfully used word embedding algorithms borrowed from natural language processing for assigning functions to genes lacking a significant sequence similarity to any sequence with a known function (Miller et al. 2022). However, systematic approaches for this kind of intragenomic annotation transfer are still lacking, which limits our ability to annotate the “dark matter” of bacterial genomes at scale.

### Orthology of Genes and Gene Products

Orthology is typically identified on the protein level, because the phylogenetic signal that is necessary to infer the precise evolutionary relationships between sequences decays slower for amino acid sequences than for nucleotide sequences. This raises the question of how to deal with genes that give rise to alternatively spliced transcripts encoding different protein isoforms. Traditionally, only one isoform per gene was considered in the ortholog search. For example, there is a one-gene-one-protein layout for the reference proteomes provided by UniProt that are used for the QfO benchmark (Nevers et al. 2022). However, any prior selection of representative isoforms can impair the outcome of the ortholog search. Orthologs may be missed if a short isoform is chosen as the representative in one species and a long isoform represents the gene in another species. Alternatively, differences in the feature architecture of two orthologs (see Challenge 2) may reflect alternative splice events instead of evolutionary change. One approach to ameliorate this issue is the selection of one or several representative isoforms. The Ortho2tree pipeline, for example, provides a consistent set of canonical isoforms for closely related species using multiple sequence alignments and phylogenetic clustering (Insana et al. 2024). Other approaches additionally consider proteomic and structural data and involve human curation. However, these are currently only available for model organisms (Morales et al. 2022; Rodriguez et al. 2022). As an alternative concept, individual ortholog search tools allow for a selection on the fly by identifying the isoform that provides the best pairwise alignment scores to orthologs from all other considered species (Altenhoff et al. 2021).

Irrespective of the precise selection procedure, restricting comparative analysis to only one isoform per gene neglects the functional complexity in a proteome conveyed by these isoforms (Manuel et al. 2023) and how this has evolved. To accommodate this aspect, orthologous isoforms have to be identified, which makes it necessary to extend the concept of orthology to also include the evolutionary history of alternative splice patterns. To better compare the true functional repertoire of different proteomes, isoform-aware orthology assignment methods are required. As the first step in this direction, SplicedFamAlign introduces the concept of transcript orthologous groups. Specifically, the software analyses exon structure preservation as well as a one-to-one correspondence between the exons of two transcripts (Jammali et al. 2019).

### Pan-genome-based Orthology Inference

In the current surge of genomic sequencing, available data do not only become more broadly distributed across the tree of life. It also becomes deeper, with multiple and sometimes even thousands of individuals being sequenced per species. This wealth of data allows the focus of comparative analyses to extend from an individual genome to all genes observed in a taxonomic clade, the pan-genome. Using a single assembly as a representative for the genomic diversity of a species is common practice, but it introduces a reference bias (Eizenga et al. 2020). For example, each additional human genome sequence adds, on average, 23 Mb of euchromatic autosomal sequence to the GRCh38 assembly (Liao et al. 2023). Genes residing in these nonreference regions are consequently missed in reference-based analyses.

One powerful alternative to reference-based approaches are pan-genome graphs. They employ sequence alignments (Eizenga et al. 2020) or conservation of gene order (Gautreau et al. 2020) to represent the pan-genome in a compact data structure. This works well to capture evolutionary dynamics in the pan-genome as long as the evolutionary distances remain short. With longer distances, however, both sequence similarity and gene-order conservation decrease. Additionally, the number of gene duplications increases which introduces reticulations in the pan-genome graph. Replacing the currently employed unidirectional searches to identify corresponding genes across genomes with an orthology inference could resolve these reticulations; however, the additional computational complexity diminishes the benefit of pan-genome graphs.

Capturing the pan-genome using hierarchical orthologous groups is an alternative to the reference-based approach, which, however, ignores gene-order conservation. This idea is applied in the Microbial Genome Database for Comparative Analysis (MBGD) that constructs groups of orthologs on multiple taxonomic levels for 15,397 assemblies from 4,747 species in 1,444 genera (Uchiyama et al. 2019). This approach can cover larger evolutionary distances, but its range remains limited by the computational complexity of all-versus-all ortholog searches across gene set collections whose numbers increase in broader taxonomic scopes.

Future studies are now needed to find ways to harness the potential of integrating both gene-order conservation and orthology inference over larger evolutionary distances, while keeping the computational overhead to a minimum.

### Orthologs of noncoding RNAs

Proteins were long treated as the main agent of biological function, and consequently, the Quest for Orthologs has first started to unravel the evolutionary history of protein-coding genes. However, up to 98% of all human transcripts are noncoding RNAs (ncRNAs) (Alessio et al. 2020). Even though there has been a surge of publications that start to elucidate the functional role of these noncoding transcripts (Mattick et al. 2023), transferring new information between them via ortholog prediction has remained challenging.

The identification of orthologs to microRNAs (miRNAs) was mainly hindered by their small size (∼22 nt) and because they can be in repeat-rich regions. High-quality annotations of miRNAs require densely sampled small RNA-seq datasets and expert knowledge (Fromm et al. 2022). With the development of MirMachine, annotations of 508 miRNA families can now be extended to genome assemblies without the help of transcriptomic data (Umu et al. 2023). This is done by training a covariance model for each family with high-confidence miRNAs from MirGeneDB (Fromm et al. 2022). These models can serve as the first step of an ortholog search, in principle, but are limited to conserved miRNA families and training sequences are restricted to species represented in MirGeneDB. These limitations have been recently overcome by ncOrtho, which exploits regions of conserved synteny to first identify a set of positional miRNA orthologs in any given set of genome assemblies (Langschied et al. 2023). These high-confidence orthologs are then used for training covariance models which form the basis for a subsequent model-based ortholog search. Ortholog assignments by ncOrtho match gold-standard annotations in precision and facilitate high-resolution studies on the evolution and taxonomic distribution of miRNA families.

Predicting orthologs of other classes of noncoding RNAs remains challenging because their sequences change rapidly over time, even if their function remains conserved (Ross et al. 2021). Orthologs between species as closely related as human and mouse can be identified by considering conservation of splice sites and sites of active transcription (Chen et al. 2016). However, this is only possible if high-quality annotations, whole-genome alignments as well as transcriptomic data are available for both species. Therefore, lncRNA databases either lack orthology assignments completely (Zhou et al. 2021), are restricted to manually curated models of a few lncRNA families (Kalvari et al. 2021), or cover only a small taxonomic clade like primates (Bryzghalov et al. 2020).

The development of methods for the detection of miRNA orthologs is a first step for integrating ncRNAs into orthology-based frameworks. For the first time, miRNAs are available as markers in phylogenomic analyses, and their evolutionary dynamics can now be studied with unprecedented resolution. However, developing new methods for identifying orthologs from other classes of ncRNAs is key for continuing the Quest for Orthologs.

## Challenge 4: Integrating Orthology With Protein Structure

Multiple studies have shown that the evolutionary age of many genes is underestimated due to sensitivity limits of sequence similarity-based orthology assignments (Jain et al. 2019; Weisman et al. 2020). Given that protein structure is up to ten times more conserved than the amino acid sequence (Illergård et al. 2009), this additional level of information promises to extend orthology prediction to even deeper timescales.

In July 2021, DeepMind and the European Bioinformatics Institute released AlphaFold Database, which in its current version covers the vast majority of proteins in UniProt with over 200 million protein structure models (Varadi et al. 2024). Additionally, embeddings from protein language models have been applied in recent years for various bioinformatics tasks, including function and structure prediction, but also homology assignments (Heinzinger et al. 2022). Therefore, high-quality models of protein structures are suddenly easy to obtain and provide an excellent basis for finding evolutionary-related proteins. With the development of the ultra-fast structural aligner Foldseek (van Kempen et al. 2023), homology assignments across large evolutionary timescales are increasingly viable (Ruperti et al. 2023). Indeed, protein structure-based searches enable the discovery of homologs across the most distant branches of the tree of life. For example, the same arrangement of the spaH domain followed by a beta-propeller protein domain is observed in both eukaryotic nuclear pore complex proteins and bacterial protomembranes, despite their sequence similarity being < 4% (Santarella-Mellwig et al. 2010).

However, available strategies for structure-based homology assignments still struggle to distinguish between orthologs and paralogs. Nevertheless, predicted protein structures can identify cases in which orthologs have diverged on structure level (Iruegas et al. 2023). In this sense, protein structure serves as an additional feature such as domain architecture that can be used to infer if the function of orthologs has changed (see Challenge 2).

Applied researchers are eager to leverage similar protein structures as a means for transferring annotations of protein function, which has traditionally been based on orthology. Since structure and orthology are two different concepts, their assignments of “corresponding” proteins may clash. This happens, for example, in the case of convergent evolution of protein structures from different origins or the subfunctionalization of paralogs that is not reflected in the structure of a protein (Bordin et al. 2021). Both these concepts should therefore be used in complement to each other wherever possible, but a framework for combined utilization of these data is yet to be developed.

## Challenge 5: Making Orthology Inferences FAIR

The concept of “Green bioinformatics” emphasizes the need to avoid unnecessary computations and energy usage (Lannelongue et al. 2023). As an orthology research community, we have therefore the responsibility to be efficient and environmentally responsible. The computational and logistical effort to precompute orthology assignments across many taxa only becomes justified if a wide range of users has access to these data and uses it (Mendes de Farias et al. 2022). Making data accessible to users from different backgrounds, including software developers, researchers and the general public, is best achieved by adhering to the FAIR data principles (Wilkinson et al. 2016). The various dedicated public databases for the dissemination of orthology inferences have been a first step in this direction. Publicly available orthology relationships are summarized in DIOPT, a platform integrating the prediction results of 19 algorithms as well as the annotation effort from model organism databases, that allows users to filter orthologs based on votes and rankings, providing protein alignments, domain information, and species conservation data (Hu et al. 2011). It further gives individual users the option to adjust the strictness with which orthologs are assigned: do they want to take orthologs that are predicted by many tools and are therefore likely to be true, or do they want to cast a broad net and take all putative orthologs inferred by any given method? Being originally tailored toward the *Drosophila* community, the functionality of DIOPT was later extended to other model organisms (Hu et al. 2017) and was used mapping various datasets across species such as interaction data (Hu et al. 2018) and single-cell data (Hu et al. 2021). Other platforms like the Gene Nomenclature Committee of the Human Genome Organization (HGNC) Comparison of Orthology Predictions (HCOP) tool (Wright et al. 2005) and the Alliance of Genome Resources (The Alliance of Genome Resources Consortium 2020) also provide meta-predictions that integrate various sources of orthology assignments with functional and other additional information.

To improve accessibility, new ways of querying databases may prove useful. One possibility is to convert scientific questions to database queries. Natural language processing could enable users to query databases in a manner that more closely resembles natural language questions. This has the potential to make the accessibility of orthology databases similar to that of chatbots like ChatGPT. Additionally, federated SPARQL queries could facilitate the gathering of information between databases (Sima et al. 2019).

A further approach for reducing the environmental footprint of ortholog searches is to share resources, results, and knowledge to avoid redundant computations. The OrthoXML format for orthology data has been proposed as a standard format for sharing this information (Schmitt et al. 2011). Unfortunately, it still faces challenges in terms of compatibility and user-friendliness and more work is necessary to provide a relevant data exchange standard. To reduce the need for all-against-all computations, new tools like OMAmer (Rossier et al. 2021) and SHOOT (Emms and Kelly 2022) have been developed to assign new gene sets to orthologous groups and place query sequences onto phylogenetic trees, respectively. In this vein, the matter of cross-referencing between releases becomes increasingly important. This is especially of concern for the reconstruction of meta-databases like MetaPhOrs v2.5 (Chorostecki et al. 2020) or phylome databases like PhylomeDB v5 (Fuentes et al. 2022). Unless advancements occur in the methods employed for crosslinking information from disparate databases across different versions of a species’ proteome, a necessity to recalibrate analyses using the latest proteomes and their corresponding annotations will persist. Not addressing this challenge jeopardizes the preservation of previously documented information but also imposes an extensive computational burden.

While considerable efforts have been made thus far to make orthology accessible to the scientific community, communicating orthology-related scientific concepts to the public has lagged. Without a basic understanding of scientific concepts, the public may fall prey to harmful misinformation and sensationalism. Evolution, a foundational pillar of biology, is often misunderstood or misrepresented. Science communication projects like “In the Light of Evolution” (https://lightofevolution.org/) aim to foster curiosity about evolution and bioinformatics among people of all ages (Blatter et al. 2023). These projects create engaging stories and activities based on genuine scientific publications, offering participants tangible learning experiences. Orthology data from databases like OMA play a key role in these projects, helping to educate the public about common ancestry, relatedness, and evolution in a fun and engaging way. By providing opportunities that reflect real-world scientific practices, these initiatives provide valuable insights into the workings of scientists.

Accessibility and reusability of orthology information are essential for the orthology community. By understanding the needs of biologists, developing user-friendly tools, incorporating NLP for database querying, engaging in science communication with the public, and promoting resource sharing and green bioinformatics practices, we can make orthology more accessible and beneficial for all stakeholders.

## Conclusion

Densely sampled collections of genome assemblies allow biodiversity genomics projects to operate at new scales. The inference of evolutionary relationships forms the basis of these projects but faces several challenges that need to be addressed in future studies. Different challenges connect to different areas of biodiversity genomics (Fig. 1). For example, conservation genomics benefits most from identifying complete gene catalogues in large collections of assemblies (Challenge 1) but might be less interested in the functional differences of the orthologs therein (Challenge 2). In contrast, finding differences in molecular functions will be one of the main focuses of biomedical research. Testing orthologs for conserved domain architecture or protein structure similarity promises to improve the accuracy of any fine-grained analysis in this field (Challenge 2). Other areas like natural product genomics are impacted by each challenge presented here. Finding genes that synthesize new or specific secondary metabolites benefits from a large body of sequenced and annotated genes (Challenge 1). Changes in domain architecture or predicted protein structure might indicate functional adaptations that have an impact on the produced metabolite (Challenges 2 and 4). Biosynthetic genes may produce short proteins or remain hidden in the unannotated “bacterial dark matter” (Challenge 3). Lastly, researchers that are interested in natural product genomics must be aware of recent advances in orthology prediction and know how to apply them (Challenge 5). Currently, many different communities rely on orthology prediction implicitly because they are interested in the annotation transfer between genes. Looking ahead, we must strive to connect these communities and create more awareness of the opportunities created by the Quest for Orthologs.

## Data Availability

No new data were generated or analyzed in support of this research.

## Members of the Quest for Orthologs (QfO) Consortium

Adrian Altenhoff, Aida Ouangraoua, Alex Warwick Vesztrocy, Arnaud Kress, Christophe Dessimoz, Dannie Durand, David Emms, Diego Fuentes-Palacios, Emma Persson, Erik Sonnhammer, Felix Langschied, Ikuo Uchiyama, Ingo Ebersberger, Jaime Huerta-Cepas, Laetitia Poidevin, Luis Pedro Coelho, Maria J. Martin, Michael Hiller, Natasha Glover, Nicola Bordin, Odile Lecompte, Paul D. Thomas, Saioa Manzano-Morales, Salvador Capella-Gutierrez, Salvatore Cosentino, Silvia Prieto Baños, Sina Majidian, Sofia Kirke Forslund-Startceva, Stefano Pascarelli, Thomas A. Richards, Toni Gabaldón, Vinh Tran, Wataru Iwasaki, Yan Wang, Yannis Nevers.
